# Adverse events following immunization in pregnant women from Minas Gerais

**DOI:** 10.11606/s1518-8787.2021055002592

**Published:** 2021-05-06

**Authors:** Isabela Oliveira da Silveira, Thales Philipe Rodrigues da Silva, Bianca Maria Oliveira Luvisaro, Roberta Barros da Silva, Josianne Dias Gusmão, Aline Mendes Vimieiro, Valéria Conceição de Oliveira, Karina Cristina Rouwe de Souza, Ana Paula Vieira Faria, Fernanda Penido Matozinhos

**Affiliations:** I Universidade Federal de Minas Gerais Escola de Enfermagem Belo Horizonte MG Brasil Universidade Federal de Minas Gerais. Escola de Enfermagem. Graduação em Enfermagem. Belo Horizonte, MG, Brasil.; II Universidade Federal de Minas Gerais Programa de Pós-Graduação em Ciências da Saúde Faculdade de Medicina Belo Horizonte MG Brasil Universidade Federal de Minas Gerais. Faculdade de Medicina. Programa de Pós-Graduação em Ciências da Saúde – Saúde da Criança e do Adolescente. Belo Horizonte, MG, Brasil.; III Universidade Federal de Minas Gerais Programa de Pós-Graduação em Saúde e Enfermagem Escola de Enfermagem Belo Horizonte MG Brasil Universidade Federal de Minas Gerais. Escola de Enfermagem. Programa de Pós-Graduação em Saúde e Enfermagem. Belo Horizonte, MG, Brasil.; IV Secretária de Estado da Saúde de Minas Gerais Subsecretária de Vigilância em Saúde Superintendência de Vigilância Epidemiológica Belo Horizonte MG Brasil Secretária de Estado da Saúde de Minas Gerais. Subsecretária de Vigilância em Saúde. Superintendência de Vigilância Epidemiológica. Belo Horizonte, MG, Brasil.; V Universidade Federal de São João Del Rey Campus Centro Oeste Dona Lindu Divinópolis MG Brasil Universidade Federal de São João Del Rey. Campus Centro Oeste Dona Lindu. Grupo de atuação docente de saúde coletiva. Divinópolis, MG, Brasil.; VI Universidade Federal de Minas Gerais Escola de Enfermagem Departamento de Enfermagem Materno-Infantil e Saúde Pública Belo Horizonte MG Brasil Universidade Federal de Minas Gerais. Escola de Enfermagem. Departamento de Enfermagem Materno-Infantil e Saúde Pública. Belo Horizonte, MG, Brasil.

**Keywords:** Vaccination, adverse effects, Pregnant Women, Epidemiology, Information Systems

## Abstract

**OBJECTIVE:**

To analyze the distribution of adverse events following immunization (AEFI) in pregnant women in the state of Minas Gerais, between 2015 and 2019.

**METHODS:**

This is an epidemiological, descriptive study conducted with AEFI data from 2015 to 2019, recorded in the Adverse Events Surveillance Information System, in the state of Minas Gerais (MG), Brazil. A total of 670 AEFI were analyzed in pregnant women. The estimates were presented in proportions, according to the year of occurrence, health macro-region of Minas Gerais and immunobiological administered.

**RESULTS:**

The year in which there were the most records was 2017 (36.8%). Among the 14 macro-regions, the ones with the lowest and highest number of records were the Vale do Jequitinhonha (0.5%) and Center (31.8%), respectively. The vaccines contraindicated during pregnancy represented 27.6% of the total notifications. The total of 69.5% of the cases were considered immunization errors. In 75.9% of the records, the variable of medical care was ignored, and in 73.7% of the cases no information on the evolution was presented.

**CONCLUSION:**

This study shows the need for continuing education for the multidisciplinary team, in order to reduce cases of AEFI and ensure the adequate completion of notifications by health professionals.

## INTRODUCTION

Vaccination is a priority, effective and strategic action of Primary Health Care (PHC)^1^. Immunization programs contribute to improving quality and increasing global life expectancy by reducing, controlling or eradicating preventable immunopreventable diseases^2^. The Brazilian Immunization Program (PNI), created in 1973, is recognized worldwide for providing free access to vaccination for the entire population and for its degree of complexity, since the number of immunobiologicals offered is high, and vaccination regimens are diversified^3^.

Initially, the main target of PNI were children, and over the years IR contributed to improve the average vaccination coverage of children under 1 year of age. Throughout its trajectory to this day, the program has undergone several modifications in the vaccination schedule. Currently, the PNI covers all age groups and life cycles, such as adolescents, older adults and pregnant women^4^.

In the context of the health of Brazilian pregnant women, the vaccination schedule has been improved. Pregnant women are at risk of higher complications due to immunopreventable and potentially fatal diseases^5^, because during the gestational period, women undergo changes in the immune and physiological system that can increase susceptibility to infectious diseases^5^^,^^6^.

Vaccination during pregnancy is a vital preventive measure in routine obstetric care, serving to protect the mother, fetus and baby. Vaccines are administered according to specific vaccination schedule, based on a vaccine schedule proven effective for pregnant women. In view of the increase in the number of immunobiologicals offered and the complex management situation provided by the PNI, the occurrence of adverse events following immunization (AEFI) has also increased^7^.

AEFI are severe, undesirable or unexpected signs or symptoms manifested in the individual who has received any type of immunobiological. Such events can be caused by several factors related to immunobiological components, the vaccination process or the vaccine itself^7^^,^^8^. The AEFI are classified according to their extent — local or systemic — and intensity — mild (when there is no need for complementary tests or medical treatment); moderate (when there is a need for medical evaluation and complementary examinations or medical treatment); and severe (when it triggers hospitalization for at least 24 hours, significant or persistent dysfunction or disability, that is, sequelae, which results in a congenital anomaly or requires immediate intervention to prevent death^9^.

In 1991, the World Health Organization (WHO) recommended that surveillance of adverse events be established after vaccination. In Brazil, in 2000, the Ministry of Health, through the PNI, implemented the Information System for Surveillance of Adverse Events Following Immunization (IS-AEFI), with the objective of controlling these events through surveillance, notification, monitoring and investigation of the cases that occurred, offering subsidies to identify predictors and risk groups^9^.

A study on the influenza vaccination campaign in Cuba evaluated the occurrences and severity of AEFI in pregnant women. Only 0.8% of the research participants had AEFI, and most events were classified as mild^10^. Another study analyzed the registration of AEFI in hepatitis B vaccines in pregnant women and their perinatal repercussions in the United States between 1990 and 2016. Common AEFI stood out in relation to local reactions; there was no record of maternal death and no cases of administration error were recorded^11^.

Despite international studies^10^^–^^12^, in the Brazilian literature, studies on AEFI are still incipient. Considering the importance of these events in decision-making in health services and the magnitude of their occurrence among pregnant women, this study aimed to analyze the distribution of AEFI in pregnant women in the state of Minas Gerais, between 2015 and 2019.

## METHODS

**Figure 1:**
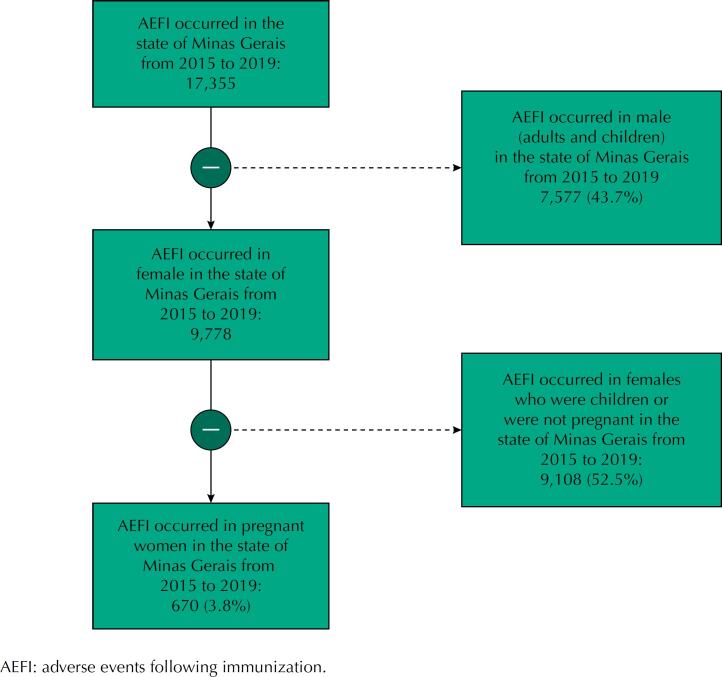
Sample selection flowchart.

This is an epidemiological, descriptive study conducted with data from the PNI Information System of the state of Minas Gerais (MG), Brazil, from January 1, 2015 to December 31, 2019. We analyzed all records of this period related to pregnant women, i.e., 670 AEFI. The selection flowchart of the AEFI sample in pregnant women can be observed in Figure 1.

The state of Minas Gerais consists of 853 municipalities, distributed in a territory of 586,522,122 km^2^, with a population of 21,168,791 inhabitants in 2019^13^^,^^14^. The state was divided into 14 health macro-regions, considered as a territorial basis for health care planning due to their demographic, socioeconomic, geographic, sanitary, epidemiological, service provision and relationships among municipalities. Namely: South; South Central; Center; Jequitinhonha; West; East; Southeast; North; Northwest; South East; Northeast; Southern Triangle; Northern Triangle; and Vale do Aço.

In this study, we analyzed the cases of AEFI with and without closure occurred in the defined period. The following variables were analyzed for the cases of AEFI: year of occurrence of the event; macro-region of health of occurrence; administered immunobiological; type of event (non-severe, severe, immunization error, immunization error with adverse event); medical care (yes, no, ignored); and evolution of the case (cure without sequelae, cure with sequelae, under follow-up, is not AEFI, death, others).

AEFI notifications were also analyzed based on the national vaccination schedule recommended for pregnant women^9^, with immunobiologicals divided into recommended vaccines during pregnancy, vaccines recommended in special situations during pregnancy and vaccines contraindicated during pregnancy. These vaccines are recommended during pregnancy: the adult-type acellular triple bacterial – diphtheria, tetanus and pertussis – (dTpa); the adult duo – diphtheria and tetanus – (dT); hepatitis B; and influenza. They are vaccines recommended in special situations during pregnancy (application is made through careful analysis of the health service, which will judge the relevance of vaccination): hepatitis A and B; pneumococcal; meningococcal ACWY/C conjugated; meningococcal B; and yellow fever. And, finally, they are vaccines contraindicated in pregnancy: triple viral (measles, mumps and rubella); human papillomavirus (HPV); chickenpox (chickenpox) and dengue.

The yellow fever vaccine is contraindicated for pregnant women, however, in the impossibility of postponing vaccination, such as in epidemiological emergencies, outbreaks or epidemics, the health service should assess the relevance of vaccination. In the state of Minas Gerais, in 2017 and 2018, there were epidemiological outbreaks of yellow fever, and unvaccinated pregnant women living in areas with active transmission of the disease received a dose of the vaccine (in any gestational period)^15^. For this reason, this immunobiological was considered as recommended in special situations during pregnancy.

The incidence rate (IR) of adverse events per 100,000 doses applied was also estimated. For the estimation of IR, the number of AEFI in pregnant women was considered in the numerator and, as denominator, the number of doses administered in pregnant women in the period, by health macro-region. The number of doses was obtained on the website of the Ministry of Health (pni.datasus.gov.br). In the search, the filter available for age group (10 to 49 years) was used, considering only the application in pregnant women.

For data analysis, the Statistical Software for Professional (Stata) program, version 14.0, was used. The estimates of the AEFI were presented in proportions (%), with their respective confidence intervals (95%CI) according to the year of occurrence, health macro-region and immunobiological administered. It is emphasized that the number of immunobiologicals administered is different from the number of pregnant women, because a single pregnant woman may have received more than one immunobiological in the gestational period. For the age of the pregnant women, due to the asymmetry in the evaluation by the Shapiro-Wilk test, the data were presented by means of median and interquartile range (IQR).

The research, under the title “Vaccination of pregnant women: evaluation of epidemiological and clinical aspects in the city of Belo Horizonte,” was approved by the Ethics Committee of the Universidade Federal de Minas Gerais, under the protocol CAAE 53843716.0.0000.5149.

## RESULTS

From 2015 to 2019, 17,355 AEFI were registered in the state of Minas Gerais; among them, 670 (3.8%) occurred in pregnant women. The median age of pregnant women who presented such events was 28.18 years (IQR: 22.56–33.18). Regarding self-reported skin color, 42.7% of the notifications did not bring this information. Among the 384 (57.3%) pregnant women who had self-reported skin color, 54.9% were yellow/brown, 34.4% white, and 10.7% black.

**Figure 2:**
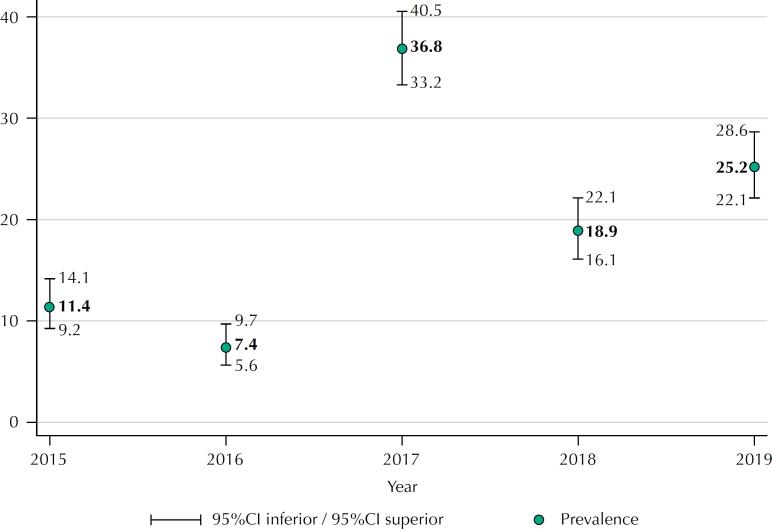
Adverse events following immunization in pregnant women (%) and 95%CI, second year of occurrence (Minas Gerais, Brazil, 2015 to 2019).

During the period from 2015 to 2016, the number of cases of AEFI in pregnant women fluctuated from 7.4% (95%CI: 5.6%–9.7%) (50 cases) in 2016, at 36.8% (95%CI: 33.2%–40.5%) (247 cases) in 2017 (Figure 2).

The data presented in Table 1 show the AEFI by the health macro-regions. The Center macro-region, with the highest number of pregnant women in the state from 2015 to 2019 (32.9% of the total), recorded the highest proportion (31.8%) AEFI. The macro-region with the lowest proportion was Jequitinhonha, with 0.5%.

**Table 1:** Incidence rate of adverse event following immunization in pregnant women per 100,000 doses applied, according to health macro-regions (Minas Gerais, Brazil, 2015 to 2019).

Health macro-regions	Population of pregnant women^a^	%^b^ of the total population of pregnant women in the state	Doses applied in pregnant women^c^	n^d^	TI^e^	%^b^ of total AEFI^f^
Center	320,536	32.9	293,977	213	72.5	31.8
South-Central	32,952	3.4	25,937	9	34.7	1.3
Jequitinhonha	18,886	1.9	13,284	3	22.6	0.5
East	32,641	3.3	27,427	8	29.2	1.2
South East	38,132	3.9	25,902	20	77.2	3.0
Northeast	38,973	4.0	30,195	9	29.8	1.3
Northwest	32,687	3.4	26,149	8	30.6	1.2
North	83,558	8.6	75,950	29	38.2	4.3
West	54,872	5.6	46,413	32	68.9	4.8
Southeast	63,465	6.5	70,176	24	34.2	3.6
South	121,403	12.5	95,528	91	95.3	13.6
Northern Triangle	61,911	6.3	64,937	61	93.9	9.1
Southern Triangle	36,556	3.7	30,299	47	155.1	7.0
Vale do Aço	38,495	3.9	44,896	116	258.4	17.3
Total	975,067	100.00	871,070	670	76.9	100.00

%: relative frequency;

a population of pregnant women from 2015 to 2019;

b relative frequency;

c the number of doses was obtained by the Website of the Ministry of Health (pni.datasus.gov.br) and considered only the application in pregnant women;

d sample number;

e IR: incidence rate of adverse event following immunization in pregnant women per 100,000 doses applied;

f AEFI: adverse events following immunization.

The incidence rate of AEFI for pregnant women, by health macro-regions, can also be observed in Table 1. The macro-region with the highest rate of adverse events after vaccination was that of Vale do Aço, where 116 EAFI were reported, an incidence rate of 258.4 cases per 100,000 doses administered in pregnant women. The macro-region with the lowest incidence rate was the Jequitinhonha macro-region, with 22.6 AEFI per 100,000 doses applied.

Data on administered immunobiologicals can be seen in Table 2. We found that the highest proportion (39.9%) recommended vaccines during pregnancy. However, 27.7% of the events related to vaccines contraindicated in the gestational period. The immunobiological with the highest proportion of AEFI (31.9%) in pregnant women it was the yellow fever vaccine, followed by the triple viral (23.6%), contraindicated during pregnancy.

**Table 2:** An adverse event following immunization in pregnant women, according to the recommendation of the Ministry of Health for the gestational and immunobiological period (Minas Gerais, Brazil, 2015 to 2019).

Immunobiologicals		n (%)^a^	n (%)^b^
Violence during pregnancy		317 (39.9)	317(100)
	Adult duo	89 (11.2)	89 (28.1)
	Influenza	43 (5.4)	43 (13.6)
	Hepatitis B	66 (8.3)	66 (20.8)
	Acellular bacterial triple (adult) — dTpa	119 (15.0)	119 (37.5)
Recommended in special situations		257 (32.4)	257 (100)
	Yellow fever	253 (31.9)	253 (98.4)
	Meningococcal conjugated C	2 (0.3)	2 (0.8)
	Rabies in Vero cell culture	2 (0.3)	2 (0.8)
Contraindicated		220 (27.7)	220 (100)
	DTP/HB/Hib	6 (0.8)	6 (2.7)
	Quadrivalent HPV	13 (1.6)	13 (5.9)
	Tetra viral	1 (0.1)	1 (0.5)
	Triple bacterial DTP	13 (1.6)	13 (5.9)
	Triple viral	187 (23.6)	187 (85.0)
Total		794 (100)	

%: relative frequency;

a % estimated for all immunobiologicals applied;

b % estimated in relation to the indication of administration.

Regarding the vaccines recommended for pregnant women, we observed that the acellular bacterial triple (dTpa) was the one that presented the most AEFI (37.5%), followed by the adult duo (dT), with 28.1%. Among the immunobiologicals recommended in special situations, the yellow fever vaccine accounted for 98.4% of all AEFI in this category. As for vaccines contraindicated during pregnancy, the triple viral vaccine presented 85% of the events in this category (Table 2).

Considering the characteristics of the AEFI, we observed that 69.5% of the cases were classified as immunization error (Table 4). There was medical care in 14.6% of the reported cases; however, in 76% of the AEFI records, the completion of this variable was ignored. Of the total of 670 AEFI, 17.3% had cure without sequelae, and the prevalence of records without information on the evolution of the case was 73.7% (Table 3).

**Table 3:** Characteristics of adverse events following immunization among pregnant women, second year of occurrence (Minas Gerais, Brazil, 2015 to 2019).

Type of event		Year					Total
		2015	2016	2017	2018	2019	
		n (%)	n (%)	n (%)	n (%)	n (%)	n (%)
Immunization error		30 (39.9)	25 (50.0)	199 (80.6)	90 (70.9)	122 (72.2)	466 (69.5)
	Immunization error with adverse event	2 (2.6)	3 (6.0)	5 (2.0)	4 (3.2)	4 (2.4)	18 (2.7)
	Severe	1 (1.3)	2 (4.0)	3 (1.2)	–	–	6 (0.9)
	non-severe	44 (57.2)	20 (40.0)	40 (16.2)	33 (25.9)	43 (25.4)	180 (26.9)
Medical care							
	Ignored	34 (44.2)	27 (54.0)	210 (85.0)	97 (76.4)	141 (83.4)	509 (76.0)
	No	19 (24.7)	9 (18.0)	14 (5.7)	11 (8.7)	10 (5.9)	63 (9.4)
	Yes	24 (31.1)	14 (28.0)	23 (9.3)	19 (14.9)	18 (10.7)	98 (14.6)
Evolution of the case							
	Healing with sequelae	–	–	1 (0.4)	2 (1.6)	–	3 (0.4)
	Healing without sequelae	38 (49.4)	15 (30.0)	23 (9.3)	16 (12.6)	24 (14.2)	116 (17.3)
	Under follow-up	8 (10.4)	7 (14.0)	14 (5.7)	13 (10.2)	4 (2.4)	46 (6.9)
	It is not AEFI	1 (1.3)	1 (2.0)	4 (1.6)	4 (3.2)	–	10 (1.5)
	Lost to follow-up	–	–	–	–	1 (0.6)	1 (0.2)
	No information	30 (38.9)	27 (54.0)	205 (83.0)	92 (72.4)	140 (82.8)	494 (73.7)

%: relative frequency; AEFI: adverse event following immunization.

Finally, among the three pregnant women (0.4%) who had a cure with some type of sequelae, we observed that the median age was 33 years (IQR: 24–57). Regarding the immunobiological administered, each pregnant woman received a distinct vaccine: triple viral vaccine, trivalent influenza and dTpa. Two pregnant women required medical attention. She was hospitalized for 2 to 3 days, and one of them, who had received the triple viral, had an abortion.

## DISCUSSION

The results of this study showed that the distribution of AEFI in pregnant women in the state of Minas Gerais occurred without a determined trend between the years considered, with a higher incidence in 2017. The macro-region with the highest percentage of AEFI notification was that of the Center, and the macro-region with the highest incidence rate was the Vale do Aço. The vaccines with the highest proportion of AEFI registration were those not recommended in the national immunization calendar, with yellow fever and triple viral being the largest responsible for increasing this number. Immunization error was the most prevalent type of event in the population, and 73.7% of the cases did not have the evolution of the case reported.

Among the years studied, the ones with the lowest incidence of notifications were those of 2015 and 2016, with great disparity in relation to the others. The still recent support to the Online Module of The IS-AEFI, implemented in 2014, may have influenced the low amount of completion of the records. Despite reducing the inconsistencies presented by the offline mode, the change requires adaptation of the vaccinators^9^.

Regarding the number and incidence rate of AEFI in macro-regions, we can infer that there is influence of variation in the structure and prenatal care of primary care between localities, as observed in other studies^16^. Thus, factors closely linked to the occurrence and notification of AEFI can be highlighted, such as availability of materials, work process and provision of guidance to pregnant women and companions^16^. Concomitantly, other factors may be related to this variation, such as low adhering to the recording of these events, lack of information on the importance of notifying them and lack of adequate training of health professionals to perform notifications^8^.

Regarding the type of immunobiological, the yellow fever vaccine was the main responsible for AEFI: 31.78% of cases. In 2017, as already mentioned, there was an outbreak of the disease in Minas Gerais, and therefore the Ministry of Health launched new recommendations for vaccination – which led to the increase in vaccinated pregnant women^9^^,^^17^. Normally, live virus vaccines are not recommended during pregnancy, only in special cases, when the risk of illness overlaps the theoretical vaccination risk^9^^,^^18^.

The second immunobiological with the highest proportion of notification was the triple viral, contraindicated during pregnancy. The application may have occurred due to the fact that the woman is not aware of the pregnancy at the time. It is known that this vaccine is indicated in adulthood if the individual has not been immunized in childhood and is an important recommendation for women who wish to become pregnant. In this sense, the high rate of AEFI may be related to a large number of applications of childbearing age, unlike tetra viral vaccines and HPV, which are also contraindicated^9^^,^^19^. The latter are not offered by the Unified Health System (SUS) in adulthood, although the high pregnancy rates among Brazilian adolescents should be highlighted: 68.4 per 1,000 adolescents, above the global average (46 per 1,000) and Latin America (65.5 per 1,000)^20^. DTP/HB/Hib (pentavalent) and triple bacterial vaccines are recommended exclusively in childhood^7^^,^^9^.

Regarding the type of adverse event, most cases were classified as non-severe, evolving to cure without sequelae or greater harm to patients. The data confirm previous studies, which show that the benefits of the prevention of diseases of the fetus through vaccines outweigh the risks of possible adverse effects^11^^,^^17^. In addition, a Portuguese study confirmed there were no indications of specific events associated with the application of vaccines contraindicated during pregnancy. However, studies such as this are still limited and cannot give safety to the administration of these vaccines^21^.

Immunization errors accounted for 69.55% of the AEFI recorded, confirming that such errors are the major responsible for AEFI^22^. These can be considered as any preventable event, resulting from failures in the preparation, handling, storage or administration of immunobiologicals, in order to reduce or cancel the expected vaccine effect^22^^,^^23^. Immunization errors are classified as: error in production (failure to comply with good manufacturing practices that can lead to a deviation of quality, such as power changes and increased reatogenicity); error in the cold network (vaccine transported or stored incorrectly); handling error; and error in administration (non-sterile injection, reconstitution error, injection at the incorrect site, contraindication ignored, vaccine out of date), which occur due to non-compliance with standards and techniques, which may result in an adverse event^7^^,^^23^.

In the high proportion of immunization errors, the work overload of vaccinators can have great influence, the devaluation reported by professionals^24^ and, it is assumed, the fact that the woman or professional is not aware of the ongoing pregnancy at the time of immunobiological administration. Therefore, the need for continuing education is emphasized, in order to update the knowledge of the multidisciplinary team about the vaccination schedule for pregnant women recommended by the PNI. This resource is necessary in view of the large number of vaccines offered by SUS and the constant updating of the national vaccination calendar^9^.

Also as a result of this study, it is noteworthy that more than half of the notified events were closed without information, which indicates failures in filling out notifications. These gaps make it difficult to verify the interference of other factors related to AEFI in pregnant women. Greater supervision and monitoring of professionals who fill out the notification in the IS-AEFI could improve the quality of the database^25^.

Like any epidemiological study, ours has some limitations. The research was developed based on data from secondary banks, limited to specific information. Moreover, we point out that some forms were not filled out properly. It is also noteworthy the non-inclusion of AEFI notifications made in the Notification System in Sanitary Surveillance (NOTIVISA), used by private vaccination services.

## CONCLUSION

Although most of the cases registered are not severe, the discussion about such records is important to adapt the conduct of multidisciplinary team professionals regarding risk assessment in the immunization process of pregnant women. Thus, professionals can act more safely, also transmitting it to pregnant women, babies and family members.

Another important aspect concerns the need to intensify continuing education for health professionals, especially in order to improve knowledge about the vaccination schedule of pregnant women and so that the possibility of pregnancy is always investigated before the administration of the vaccine. In addition, from the greater use of AEFI records, it is possible to broaden the understanding of pregnant women about the need for vaccination and about possible adverse events, as well as sensitize professionals to greater attention to the adequate and complete filling of the records and greater commitment to notify the adverse event in the IS-AEFI, contributing to patient safety.
