# Supplementary material for: Adverse events following immunization in pregnant women from Minas Gerais
Source: Rev Saude Publica. 2021 May 6;55:24. doi: 10.11606/s1518-8787.2021055002592 (PMC8102025; doi:10.11606/s1518-8787.2021055002592)
Supplement: Translation

# Translation: Eventos adversos pós-vacinação em gestantes de Minas Gerais

**Authors:** Isabela Oliveira da Silveira, Thales Philipe Rodrigues da Silva, Bianca Maria Oliveira Luvisaro, Roberta Barros da Silva, Josianne Dias Gusmão, Aline Mendes Vimieiro, Valéria Conceição de Oliveira, Karina Cristina Rouwe de Souza, Ana Paula Vieira Faria, Fernanda Penido Matozinhos

**Affiliations:** I Belo Horizonte MG Brasil Universidade Federal de Minas Gerais. Escola de Enfermagem. Graduação em Enfermagem. Belo Horizonte, MG, Brasil.; II Belo Horizonte MG Brasil Universidade Federal de Minas Gerais. Faculdade de Medicina. Programa de Pós-Graduação em Ciências da Saúde – Saúde da Criança e do Adolescente. Belo Horizonte, MG, Brasil.; III Belo Horizonte MG Brasil Universidade Federal de Minas Gerais. Escola de Enfermagem. Programa de Pós-Graduação em Saúde e Enfermagem. Belo Horizonte, MG, Brasil.; IV Belo Horizonte MG Brasil Secretária de Estado da Saúde de Minas Gerais. Subsecretária de Vigilância em Saúde. Superintendência de Vigilância Epidemiológica. Belo Horizonte, MG, Brasil.; V Divinópolis MG Brasil Universidade Federal de São João Del Rey. Campus Centro Oeste Dona Lindu. Grupo de atuação docente de saúde coletiva. Divinópolis, MG, Brasil.; VI Belo Horizonte MG Brasil Universidade Federal de Minas Gerais. Escola de Enfermagem. Departamento de Enfermagem Materno-Infantil e Saúde Pública. Belo Horizonte, MG, Brasil.

**Keywords:** Vacinação, Efeitos Adversos, Gestantes, Epidemiologia, Sistemas de Informação

## Abstract

**OBJETIVO:**

Analisar a distribuição dos eventos adversos pós-vacinação (EAPV) em gestantes do estado de Minas Gerais, entre 2015 e 2019.

**MÉTODOS:**

Estudo epidemiológico, descritivo, realizado com dados de EAPV, do período de 2015 a 2019, registrados no Sistema de Informação da Vigilância de Eventos Adversos, no estado de Minas Gerais (MG), Brasil. Foram analisados 670 EAPV em gestantes. As estimativas foram apresentadas em proporções, de acordo com o ano de ocorrência, macrorregião de saúde de Minas Gerais e imunobiológico administrado.

**RESULTADOS:**

O ano em que mais houve registros foi 2017 (36,8%). Entre as 14 macrorregiões, as com menor e maior número de registros foram o Vale do Jequitinhonha (0,5%) e o Centro (31,8%), respectivamente. As vacinas contraindicadas durante a gestação representaram 27,6% do total de notificações. Foram considerados como erros de imunização 69,5% dos casos. Em 75,9% dos registros, a variável de atendimento médico foi ignorada, e em 73,7% dos casos não foi apresentada informação sobre a evolução.

**CONCLUSÃO:**

Este estudo expõe a necessidade da educação continuada para a equipe multidisciplinar, a fim de reduzir casos de EAPV e garantir o preenchimento adequado das notificações pelos profissionais de saúde.

## INTRODUÇÃO

A vacinação é uma ação prioritária, efetiva e estratégica da Atenção Primária à Saúde (APS)^1^. Os programas de imunização contribuem para melhorar a qualidade e aumentar a expectativa de vida mundial, ao reduzir, controlar ou erradicar doenças evitáveis imunopreviníveis^2^. O Programa Nacional de Imunizações (PNI), criado no Brasil em 1973, é reconhecido mundialmente por fornecer acesso gratuito à vacinação para a toda a população e por seu grau de complexidade, uma vez que o número de imunobiológicos ofertados é alto, e os esquemas vacinais são diversificados^3^.

Inicialmente, o PNI tinha como alvo principal as crianças, e com o passar dos anos contribuiu para melhorar a cobertura vacinal média de menores de 1 ano de idade. Ao longo de sua trajetória até os dias atuais, o programa passou por várias modificações no calendário vacinal. Atualmente, o PNI abrange todas as faixas etárias e ciclos de vida, como adolescentes, idosos e gestantes^4^.

No contexto da saúde das gestantes brasileiras, o calendário vacinal vem sendo aprimorado. Gestantes têm risco de complicações mais elevado, devido a doenças imunopreveníveis e potencialmente fatais^5^, pois, durante o período gestacional, as mulheres passam por alterações do sistema imunológico e fisiológico que podem aumentar a suscetibilidade a doenças infecciosas^5^^,^^6^.

A vacinação durante a gravidez é uma medida preventiva vital nos cuidados obstétricos de rotina, servindo para proteger a mãe, o feto e o bebê. As vacinas são administradas de acordo com calendário de vacinação específico, baseado em um esquema vacinal comprovadamente eficaz para as gestantes. Diante do aumento do número de imunobiológicos ofertado e do quadro complexo de administração disponibilizado pelo PNI, cresceu também a ocorrência de eventos adversos pós-vacinação (EAPV)^7^.

Os EAPV são sinais ou sintomas graves, indesejáveis ou inesperados, manifestados no indivíduo que tenha recebido qualquer tipo de imunobiológico. Tais eventos podem ser causados por vários fatores relacionados aos componentes do imunobiológico, ao processo de vacinação ou ao próprio vacinado^7^^,^^8^. Os EAPV são classificados segundo sua extensão – locais ou sistêmicos – e intensidade – leve (quando não há necessidade de exames complementares ou tratamento médico); moderado (quando há necessidade de avaliação médica e exames complementares ou tratamento médico); e grave (quando desencadeia hospitalização por pelo menos 24 horas, disfunção ou incapacidade significativa ou persistente, isto é, sequela, que resulta em anomalia congênita ou requer intervenção imediata para evitar o óbito^9^.

Em 1991, a Organização Mundial da Saúde (OMS) recomendou que se estabelecesse a vigilância de eventos adversos pós-vacinação. No Brasil, em 2000, o Ministério da Saúde, por meio do PNI, implantou o Sistema de Informação da Vigilância de Eventos Adversos Pós-Vacinação (SI-EAPV), com o objetivo de controlar esses eventos por meio de vigilância, notificação, monitoramento e investigação dos casos ocorridos, oferecendo subsídios para identificar preditores e grupos de risco^9^.

Um estudo sobre a campanha de vacinação contra influenza em Cuba avaliou as ocorrências e a gravidade dos EAPV em gestantes. Somente 0,8% das participantes da pesquisa apresentaram EAPV, e a maioria dos eventos foi classificada como leve^10^. Outra pesquisa analisou o registro de EAPV nas vacinas contra hepatite B em gestantes e suas repercussões perinatais nos Estados Unidos, entre 1990 e 2016. Os EAPV comuns se sobressaíram em relação às reações locais; não houve registro de morte materna e não foram registrados casos de erro de administração^11^.

Apesar de estudos internacionais^10^^–^^12^, na literatura brasileira os trabalhos sobre EAPV ainda são incipientes. Considerando a importância desses eventos na tomada de decisões nos serviços de saúde e a magnitude de sua ocorrência entre gestantes, este estudo objetivou analisar a distribuição dos EAPV em gestantes do estado de Minas Gerais, entre 2015 e 2019.

## MÉTODOS

Trata-se de um estudo epidemiológico, descritivo, realizado com dados do Sistema de Informação do PNI do estado de Minas Gerais (MG), Brasil, no período de 1º de janeiro de 2015 a 31 de dezembro de 2019. Foram analisados todos os registros desse período relativos a gestantes, ou seja, 670 EAPV. O fluxograma de seleção da amostra dos EAPV em gestantes pode ser observado na Figura 1.

**Figura 1:** Fluxograma de seleção da amostra.

O estado de MG é constituído por 853 municípios, distribuídos em um território de 586.522,122 km^2^, com população de 21.168.791 habitantes no ano de 2019^13^^,^^14^. O estado foi dividido em 14 macrorregiões de saúde, consideradas como base territorial para o planejamento da atenção à saúde em função de suas características demográficas, socioeconômicas, geográficas, sanitárias, epidemiológicas, oferta de serviços e relações entre municípios. São elas: Sul; Centro-Sul; Centro; Jequitinhonha; Oeste; Leste; Sudeste; Norte; Noroeste; Leste do Sul; Nordeste; Triângulo do Sul; Triângulo do Norte; e Vale do Aço.

Neste estudo, foram analisados os casos de EAPV com e sem encerramento ocorridos no período definido. As seguintes variáveis foram analisadas para os casos de EAPV: ano de ocorrência do evento; macrorregião de saúde de ocorrência; imunobiológico administrado; tipo de evento (não grave, grave, erro de imunização, erro de imunização com evento adverso); atendimento médico (sim, não, ignorado); e evolução do caso (cura sem sequelas, cura com sequelas, em acompanhamento, não é EAPV, óbito, outros).

As notificações de EAPV também foram analisadas com base no calendário nacional de vacinação recomendado para as gestantes^9^, sendo os imunobiológicos divididos em vacinas recomendadas durante a gestação, vacinas recomendadas em situações especiais durante a gestação e vacinas contraindicadas na gestação. São vacinas recomendadas durante a gestação: a tríplice bacteriana acelular do tipo adulto – difteria, tétano e coqueluche – (dTpa); a dupla adulto – difteria e tétano –(dT); a de hepatite B; e a de influenza. São vacinas recomendadas em situações especiais durante a gestação (aplicação é feita mediante cautelosa análise do serviço de saúde, que julgará a pertinência da vacinação): hepatites A e B; pneumocócicas; meningocócicas conjugadas ACWY/C; meningocócica B; e febre amarela. E, por fim, são vacinas contraindicadas na gestação: tríplice viral (sarampo, caxumba e rubéola); papiloma vírus humano (HPV); varicela (catapora) e dengue.

A vacina contra febre amarela está contraindicada para as gestantes, no entanto, na impossibilidade de adiar a vacinação, como em situações de emergência epidemiológica, surtos ou epidemias, o serviço de saúde deverá avaliar a pertinência da vacinação. No estado de Minas Gerais, em 2017 e 2018, houve surtos epidemiológicos de febre amarela, e as gestantes não vacinadas, residentes em áreas com transmissão ativa da doença, receberam uma dose da vacina (em qualquer período gestacional)^15^. Por esse motivo esse imunobiológico foi considerado como recomendado em situações especiais durante a gestação.

Calculou-se, ainda, a taxa de incidência de eventos adversos por 100 mil doses aplicadas. Para o cálculo da TI, no numerador foi considerado o número de EAPV em gestantes e, como denominador, o número de doses administradas em gestantes no período, por macrorregião de saúde. O número de doses foi obtido no site do Ministério da Saúde (pni.datasus.gov.br). Na busca, utilizou-se o filtro disponível para faixa etária (10 a 49 anos), considerando somente a aplicação em gestantes.

Para a análise dos dados, foi utilizado o programa *Statistical Software for Professional (Stata),* versão 14.0. As estimativas dos EAPV foram apresentadas em proporções (%), com seus respectivos intervalos de confiança (IC95%) de acordo com o ano de ocorrência, macrorregião de saúde e imunobiológico administrado. Ressalta-se que o número de imunobiológico administrado é diferente do número de gestantes, pois uma única gestante pode ter recebido mais de um imunobiológico no período gestacional. Para a idade das gestantes, devido à assimetria na avaliação pelo teste de Shapiro-Wilk, os dados foram apresentados por meio de mediana e intervalo interquartílico (IQ).

A pesquisa, com o título “Vacinação de gestantes: avaliação dos aspectos epidemiológicos e clínicos no município de Belo Horizonte”, foi aprovada pelo Comitê de Ética da Universidade Federal de Minas Gerais, sob o protocolo CAAE 53843716.0.0000.5149.

## RESULTADOS

No período de 2015 a 2019, 17.355 EAPV foram registrados no estado de MG; dentre eles, 670 (3,8%) ocorreram em gestantes. A mediana da idade das gestantes que apresentaram tais eventos foi de 28,18 anos (IQ: 22,56–33,18). Em relação à cor de pele autodeclarada, 42,7% das notificações não traziam essa informação. Entre as 384 (57,3%) gestantes que tiveram a cor de pele autodeclarada informada, 54,9% eram amarelas/pardas, 34,4% brancas, e 10,7% pretas.

Durante o período de 2015 a 2016, o número de casos de EAPV em gestantes oscilou de 7,4% (IC95%: 5,6%–9,7%) (50 casos) em 2016, a 36,8% (IC95%: 33,2%–40,5%) (247 casos) em 2017 (Figura 2).

**Figura 2:** Eventos adversos pós-vacinação em gestantes (%) e IC95%, segundo ano de ocorrência (Minas Gerais, Brasil, 2015 a 2019).

Os dados apresentados na Tabela 1 demonstram os EAPV pelas macrorregiões de saúde. Observou-se que a macrorregião Centro, com o maior número de gestantes do estado no período de 2015 a 2019 (32,9% do total), registrou a maior proporção (31,8%) de EAPV. A macrorregião que apresentou a menor proporção foi a do Jequitinhonha, com 0,5%.

**Tabela 1:** Taxa de incidência de evento adverso pós-vacinação em gestantes por 100 mil doses aplicadas, segundo macrorregiões de saúde (Minas Gerais, Brasil, 2015 a 2019).

Macrorregiões de saúde	População de gestantes^a^	%^b^ do total da população de gestantes do estado	Doses aplicada em gestantes^c^	n^d^	TI^e^	%^b^ do total de EAPV^f^
Centro	320.536	32,9	293.977	213	72,5	31,8
Centro-Sul	32.952	3,4	25.937	9	34,7	1,3
Jequitinhonha	18.886	1,9	13.284	3	22,6	0,5
Leste	32.641	3,3	27.427	8	29,2	1,2
Leste do Sul	38.132	3,9	25.902	20	77,2	3,0
Nordeste	38.973	4,0	30.195	9	29,8	1,3
Noroeste	32.687	3,4	26.149	8	30,6	1,2
Norte	83.558	8,6	75.950	29	38,2	4,3
Oeste	54.872	5,6	46.413	32	68,9	4,8
Sudeste	63.465	6,5	70.176	24	34,2	3,6
Sul	121.403	12,5	95.528	91	95,3	13,6
Triângulo do Norte	61.911	6,3	64.937	61	93,9	9,1
Triângulo do Sul	36.556	3,7	30.299	47	155,1	7,0
Vale do Aço	38.495	3,9	44.896	116	258,4	17,3
Total	975.067	100,00	871.070	670	76,9	100,00

%: frequência relativa;

a população de gestante de 2015 a 2019;

b frequência relativa;

c o número de doses foi obtido pelo site do Ministério da Saúde (pni.datasus.gov.br) e considerou somente a aplicação em gestantes;

d número amostral;

e TI: taxa de incidência de evento adverso pós-vacinação em gestantes por 100 mil doses aplicadas;

f EAPV: eventos adversos pós-vacinação.

A taxa de incidência de EAPV para as gestantes, por macrorregiões de saúde, também pode ser observada na Tabela 1. A macrorregião com maior taxa de evento adverso pós-vacinação foi a do Vale do Aço, onde foram notificados 116 EAPV, uma taxa de incidência de 258,4 casos a cada 100 mil doses administradas em gestantes. A macrorregião com menor taxa de incidência foi a macrorregião do Jequitinhonha, com 22,6 EAPV a cada 100 mil doses aplicadas.

Os dados referentes aos imunobiológicos administrados podem ser observados na Tabela 2. Verificou-se que a maior proporção (39,9%) de EAPV foi de vacinas recomendadas durante a gestação. Entretanto, 27,7% dos eventos diziam respeito a vacinas contraindicadas no período gestacional. O imunobiológico que apresentou maior proporção de EAPV (31,9%) nas gestantes foi o da vacina contra febre amarela, seguido da tríplice viral (23,6%), contraindicada durante a gestação.

**Tabela 2:** Evento adverso pós-vacinação em gestantes, segundo recomendação do Ministério da Saúde para o período gestacional e imunobiológico (Minas Gerais, Brasil, 2015 a 2019).

Imunobiológicos		n (%)^a^	n (%)^b^
Recomendados durante a gestação		**317 (39,9)**	317(100)
	Dupla adulto	89 (11,2)	89 (28,1)
	Influenza	43 (5,4)	43 (13,6)
	Hepatite B	66 (8,3)	66 (20,8)
	Tríplice bacteriana acelular (adulto) – dTpa	119 (15,0)	119 (37,5)
Recomendados em situações especiais		**257 (32,4)**	257 (100)
	Febre amarela	253 (31,9)	253 (98,4)
	Meningocócica conjugada C	2 (0,3)	2 (0,8)
	Raiva em cultivo celular Vero	2 (0,3)	2 (0,8)
Contraindicadas		**220 (27,7)**	220 (100)
	DTP/HB/Hib	6 (0,8)	6 (2,7)
	HPV Quadrivalente	13 (1,6)	13 (5,9)
	Tetra viral	1 (0,1)	1 (0,5)
	Tríplice bacteriana DTP	13 (1,6)	13 (5,9)
	Tríplice viral	187 (23,6)	187 (85,0)
Total		794 (100)	

%: frequência relativa;

a % calculada em relação a todos os imunobiológicos aplicados;

b % calculada em relação à indicação de administração.

Em relação às vacinas preconizadas para as gestantes, observou-se que a tríplice bacteriana acelular (dTpa) foi a que mais apresentou EAPV (37,5%), seguida da dupla adulto (dT), com 28,1%. Entre os imunobiológicos recomendados em situações especiais, a vacina contra a febre amarela foi responsável por 98,4% de todos os EAPV desta categoria. Quanto às vacinas contraindicadas durante a gestação, a vacina tríplice viral apresentou 85% dos eventos desta categoria (Tabela 2).

Considerando as características dos EAPV, observou-se que 69,5% dos casos foram classificados como erro de imunização (Tabela 4). Houve atendimento médico em 14,6% dos casos notificados; entretanto, em 76% dos registros de EAPV o preenchimento desta variável foi ignorada. Do total de 670 EAPV, 17,3% tiveram cura sem sequelas, e a prevalência de registros sem informação sobre a evolução do caso foi de 73,7% (Tabela 3).

**Tabela 3:** Características dos eventos adversos pós-vacinação entre gestantes, segundo ano de ocorrência (Minas Gerais, Brasil, 2015 a 2019).

		Ano					Total
		2015	2016	2017	2018	2019	
		n (%)	n (%)	n (%)	n (%)	n (%)	n (%)
Tipo de evento							
	Erro de imunização	30 (39,9)	25 (50,0)	199 (80,6)	90 (70,9)	122 (72,2)	466 (69,5)
	Erro de imunização com evento adverso	2 (2,6)	3 (6,0)	5 (2,0)	4 (3,2)	4 (2,4)	18 (2,7)
	Grave	1 (1,3)	2 (4,0)	3 (1,2)	–	–	6 (0,9)
	Não grave	44 (57,2)	20 (40,0)	40 (16,2)	33 (25,9)	43 (25,4)	180 (26,9)
Atendimento médico							
	Ignorado	34 (44,2)	27 (54,0)	210 (85,0)	97 (76,4)	141 (83,4)	509 (76,0)
	Não	19 (24,7)	9 (18,0)	14 (5,7)	11 (8,7)	10 (5,9)	63 (9,4)
	Sim	24 (31,1)	14 (28,0)	23 (9,3)	19 (14,9)	18 (10,7)	98 (14,6)
Evolução do caso							
	Cura com sequelas	–	–	1 (0,4)	2 (1,6)	–	3 (0,4)
	Cura sem sequelas	38 (49,4)	15 (30,0)	23 (9,3)	16 (12,6)	24 (14,2)	116 (17,3)
	Em acompanhamento	8 (10,4)	7 (14,0)	14 (5,7)	13 (10,2)	4 (2,4)	46 (6,9)
	Não é EAPV	1 (1,3)	1 (2,0)	4 (1,6)	4 (3,2)	–	10 (1,5)
	Perda de seguimento	–	–	–	–	1 (0,6)	1 (0,2)
	Sem informação	30 (38,9)	27 (54,0)	205 (83,0)	92 (72,4)	140 (82,8)	494 (73,7)

%: frequência relativa; EAPV: evento adverso pós-vacinação.

Por fim, dentre as três gestantes (0,4%) que tiveram cura com algum tipo de sequela, observou-se que a mediana de idade foi de 33 anos (IQ: 24–57). Em relação ao imunobiológico administrado, cada gestante havia recebido uma vacina distinta: vacina de tríplice viral, da influenza trivalente e dTpa. Duas gestantes necessitaram de atendimento médico. Ela foram internadas de 2 a 3 dias, e uma delas, que havia recebido a tríplice viral, teve o aborto como desfecho do caso.

## DISCUSSÃO

Os resultados deste estudo demonstraram que a distribuição dos EAPV em gestantes do estado de MG ocorreu sem tendência determinada entre os anos considerados, com maior incidência em 2017. A macrorregião com maior percentual de notificação de EAPV foi a do Centro, e a macrorregião com maior taxa de incidência foi a do Vale do Aço. As vacinas com maior proporção de registro de EAPV foram as não recomendadas no calendário nacional de imunização, sendo a de febre amarela e a tríplice viral as maiores responsáveis pelo incremento desse número. O erro de imunização foi o tipo de evento mais prevalente na população, e 73,7% dos casos não tiveram a evolução do caso informada.

Entre os anos estudados, os que apresentaram menor incidência de notificações foram os de 2015 e de 2016, com grande disparidade em relação aos demais. A adesão ainda recente ao módulo on-line do SI-EAPV, implantado em 2014, pode ter influenciado na baixa quantidade de preenchimento dos registros. Apesar de reduzir as inconsistências apresentadas pelo modo off-line, a mudança demanda adaptação dos vacinadores^9^.

Em relação ao número e à taxa de incidência de EAPV nas macrorregiões, pode-se inferir que há influência da variação na estrutura e no atendimento pré-natal da atenção primária entre localidades, como observado em outros estudos^16^. Assim, podem-se destacar fatores intimamente ligados à ocorrência e à notificação de EAPV, como disponibilidade de materiais, processo de trabalho e fornecimento de orientações às gestantes e aos acompanhantes^16^. Concomitantemente, podem estar relacionados a essa variação outros fatores, como baixa adesão ao registro desses eventos, falta de informação sobre a importância de notificá-los e ausência de treinamento adequado dos profissionais de saúde para realizar as notificações^8^.

No que se refere ao tipo de imunobiológico, a vacina contra a febre amarela foi a principal responsável por EAPV: 31,78% dos casos. Em 2017, como já mencionado, houve um surto da doença no estado de MG, e por isso o Ministério da Saúde lançou novas recomendações para a vacinação – o que levou ao aumento de gestantes vacinadas^9^^,^^17^. Normalmente, as vacinas com vírus vivos não são recomendadas na gestação, apenas em casos especiais, quando o risco de adoecimento se sobrepõe ao risco teórico vacinal^9^^,^^18^.

O segundo imunobiológico com maior proporção de notificação foi a tríplice viral, contraindicada durante a gestação. A aplicação pode ter ocorrido devido ao fato de a mulher não estar ciente da gravidez no momento. Sabe-se que essa vacina é indicada na fase adulta caso o indivíduo não tenha sido imunizado na infância, e constitui importante recomendação para as mulheres que desejam engravidar. Nesse sentido, o alto índice de EAPV pode estar relacionado a um grande número de aplicações em idade fértil, diferentemente das vacinas tetra viral e HPV, que também são contraindicadas^9^^,^^19^. Estas últimas não são ofertadas pelo Sistema Único de Saúde (SUS) na fase adulta, embora deva se ressaltar as altas taxas de gravidez entre adolescentes brasileiras: 68,4 por 1 mil adolescentes, taxa acima da média global (46 por 1 mil) e latino-americana (65,5 por 1 mil)^20^. As vacinas DTP/HB/Hib (pentavalente) e tríplice bacteriana são recomendadas exclusivamente na infância^7^^,^^9^.

Em relação ao tipo de evento adverso, a maior parte dos casos foram classificados como não graves, evoluindo para cura sem sequelas ou maiores danos aos pacientes. Esse dado confirma estudos prévios, que demonstram que os benefícios da prevenção de doenças do feto por meio de vacinas superam os riscos de possíveis efeitos adversos^11^^,^^17^. Além disso, estudo português confirmou não haver indícios de eventos específicos associados à aplicação de vacinas contraindicadas na gravidez. Entretanto, pesquisas como essa ainda são limitadas e não podem atribuir segurança à administração dessas vacinas^21^.

Os erros de imunização representaram 69,55% dos EAPV registrados, confirmando que tais erros são os maiores responsáveis por EAPV^22^. Estes podem ser considerados como qualquer evento evitável, proveniente de falhas na preparação, no manuseio, no armazenamento ou na administração do imunobiológico, de modo a reduzir ou anular o efeito vacinal esperado^22^^,^^23^. Os erros de imunização são classificados em: erro na produção (não cumprimento das boas práticas de fabricação que pode levar a um desvio de qualidade, como alterações de potência e aumento de reatogenicidade); erro na rede de frio (vacina transportada ou armazenada incorretamente); erro no manuseio; e erro na administração (injeção não estéril, erro de reconstituição, injeção no local incorreto, contraindicação ignorada, vacina fora do prazo de validade), que acontecem devido ao não cumprimento de normas e técnicas, podendo resultar em evento adverso^7^^,^^23^.

Na alta proporção de erro de imunização podem ter grande influência a sobrecarga de trabalho dos vacinadores, a desvalorização relatada pelos profissionais^24^ e, supõe-se, o fato de a mulher ou o profissional não estarem cientes da gestação em curso no momento da administração do imunobiológico. Enfatiza-se, portanto, a necessidade de educação continuada, visando atualizar os conhecimentos da equipe multidisciplinar acerca do calendário de vacinação para gestantes recomendado pelo PNI. Este recurso faz-se necessário diante da grande quantidade de vacinas ofertadas pelo SUS e da atualização constante do calendário nacional de vacinação^9^.

Ainda como resultado deste trabalho, destaca-se que mais da metade dos eventos notificados foram encerrados sem informação, o que indica falhas no preenchimento das notificações. Essas lacunas dificultam a verificação de interferência de outros fatores relacionados aos EAPV em gestantes. Uma maior supervisão e acompanhamento dos profissionais que preenchem a notificação no SI-EAPV poderia melhorar a qualidade do banco de dados^25^.

Como todo estudo epidemiológico, este trabalho apresenta algumas limitações. A pesquisa foi desenvolvida com base em dados de bancos secundários, limitando-se a informações específicas. Ademais, percebe-se que alguns formulários não foram preenchidos adequadamente. Destaca-se ainda a não inclusão das notificações de EAPV realizadas no Sistema de Notificação em Vigilância Sanitária (Notivisa), utilizado pelos serviços de vacinação privados.

## CONCLUSÃO

Apesar de a maioria dos casos registrados não ser grave, a discussão sobre tais registros é importante para adequar a conduta dos profissionais da equipe multidisciplinar quanto à avaliação de riscos no processo de imunização das gestantes. Assim, os profissionais podem agir com mais segurança, transmitindo-a também às gestantes, aos bebês e familiares.

Outro aspecto importante diz respeito à necessidade de intensificar a educação continuada para os profissionais da saúde, especialmente para que sejam aprimorados os conhecimentos sobre o calendário de vacinação da gestante e para que a possibilidade da gravidez seja sempre investigada antes da administração da vacina. Além disso, a partir da maior utilização dos registros de EAPV, pode-se ampliar a compreensão da gestante sobre a necessidade de vacinação e sobre possíveis eventos adversos, bem como sensibilizar os profissionais para uma maior atenção ao preenchimento adequado e completo dos registros e maior empenho em notificar o evento adverso no SI-EAPV, contribuindo para a segurança do paciente.
